# Design of Morphology-Controllable ZnO Nanorods/Nanopariticles Composite for Enhanced Performance of Dye-Sensitized Solar Cells

**DOI:** 10.3390/nano9070931

**Published:** 2019-06-28

**Authors:** Dongting Wang, Yuting Zhang, Meng Su, Ting Xu, Haizhou Yang, Shiqing Bi, Xianxi Zhang, Yuzhen Fang, Jinsheng Zhao

**Affiliations:** 1 School of Chemistry and Chemical Engineering, Shandong Provincial Key Laboratory, Liaocheng University, Liaocheng 252059, Shandong Province, China; 2 School of Chemistry and Chemical Engineering, Yulin University, Yulin 719000, Shaanxi Province, China

**Keywords:** ZnO, nanorods/nanopariticles, morphology control, dye sensitized solar cells

## Abstract

A facile one-pot approach was developed for the synthesis of ZnO nanorods (NRs)/nanoparticles (NPs) architectures with controllable morphologies. The concrete state of existence of NPs and NRs could rationally be controlled through reaction temperature manipulation, i.e., reactions occured at 120, 140, 160, and 180 °C without stirring resulted in orderly aligned NRs, disordered but connected NRs/NPs, and relatively dispersed NRs/NPs with different sizes and lengths, respectively. The as-obained ZnO nanostructures were then applied to construct photoanodes of dye-sensitized solar cells, and the thicknesses of the resultant films were controlled for performance optimization. Under an optimized condition (i.e., with a film thickness of 14.7 µm), the device fabricated with the material synthesized at 160 °C exhibited the highest conversion efficiency of 4.30% with an elevated current density of 14.50 mA·cm^−2^ and an open circuit voltage of 0.567 V. The enhanced performance could be attributed to the coordination effects of the significantly enhanced dye absorption capability arising from the introduced NPs and the intrinsic fast electron transport property of NRs as confirmed by electrochemical impedance spectroscopy (EIS) and ultraviolet–visible (UV−vis) absorption.

## 1. Introduction

Dye-sensitized solar cells (DSCs) constructed from nanostructured metal oxides such as photoanode materials are potential alternatives to conventional silicon-based photovoltaics due to their benefits of low cost, long-term stability, environmental compatibility, and ease of fabrication [1,2,3,4,5]. Since their introduction by Grätzel., great efforts have been devoted to the improvement of powder conversion efficiency (PCE) of TiO_2_–based DSCs [6]. So far, impressive efficiency reaching up to 13% has been recorded with porphyrin-based D–p–A dye used in conjunction with cobalt-based redox couple [7]. However, a major drawback of the traditional TiO_2_ nanoparticle (NP) photoanode in DSCs is the intrinsic charge recombination rate arising from sluggish electron mobility and transport properties, thus imposing an upper limit on the state-of-the-art film thickness. Underlying the background, many other oxide semiconductors with high electron transport properties have been considered and found to be promising. For instance, ZnO, Zn_2_SnO_4_, SnO_2_ and Nb_2_O_5_ have already been demonstrated as applicable materials for photoanodes [8,9,10,11,12]. In particular, ZnO has attracted widespread attention due to many remarkable advantages, such as suitable energy band position for effective electron-injection from the sensitizer and higher electron mobility (205–1000 cm^2^·V^−1^·s^−1^) in comparison with TiO_2_ (0.1–4 cm^2^·V^−1^·s ^−1^) that enables faster electron transport within the photoanode film. In addition, the ease of the anisotropic growth property makes it possible to acquire specific structures with desirable features [13,14,15]. So far, an unprecedented efficiency up to 7.5% has been reported for ZnO-based DSCs with the photoelectrode film composed of a buffer layer and multifunctional polydispersed aggregates [16]. However, the validated efficiencies of the DSC devices based on ZnO were only approximately two-fold below those based on TiO_2_, leaving plenty of operational space for improvement of the PCE through morphological and structural modifications.

Constructing well-organized nanostructures, especially one-dimensional (1-D) structures, such as nanotubes arrays, [17] nanorods (NRs), [18] and nanowires (NWs) [19] is deemed an effective route to reduce the severe charge recombination due to direct high electron transfer pathway along the peculiar nanostructures. For instance, Galoppini et al. compared the electron transport properties of the photoanodes based on ZnO NRs array and colloids, and noticed that electron transport in the device with ZnO NRs photoanode was approximately 2-fold faster than that in ZnO colloids [20]. Besides, Kretzschmar et al. observed that the electron lifetime in photoanode films fabricated with NRs was 2 times longer compared to that with nanoparticles [21]. Apart from efficient electron transport, 1-D nanostructures with tunable lengths ranging from hundreds of nanometers to micrometers are also promising light-scattering centers with enhanced optical propagation distance within the films and improved light collection efficiencies [22,23,24]. Nevertheless, excessive loss of accessible surface area and thus insufficient dye loading of these ZnO nanostructures limits their attainable photovoltaic efficiency to a relatively low level, making them less competitive in current DSCs embodiments.

Construction of the hybrid photoanode films with different nanostructures turns out to be promising for balancing the device electrical conductivity with the dye adsorption capability. Following this strategy, 1-D/0-D composite photoanodes have been designed via various methods to combine the desirable advantages of each component [25,26,27]. For example, Xiong et al. constructed ZnO composites for photoanode applications by blending the as-prepared electrospun ZnO NWs with ZnO NPs. The obtained hybrid film delivered a high conversion efficiency of 2.6%, which was almost 2-fold higher than that of cells constructed with single ZnO nanostructure [28]. Cao et al. designed ZnO NPs-NWs array hybrid photoanodes by spin-coating of colloidal dispersion of ZnO NPs on top of the prepared ZnO NW arrays perpendicularly grown on fluorine-doped tin oxide (FTO) substrate, within which NPs play the role of offering a large overall surface area for sufficient dye adsorption and NW arrays act as a direct pathway for fast electron transport. Consequently, an overall PCE of 4.2% was obtained for the NWs–NPs composite photoanode, remarkably higher than that of ZnO NWs DSC (~1.58%), suggesting the great contribution of ZnO NPs to the photovoltaic performance [29]. The aforementioned investigations all demonstrated the feasibility of significantly improving the conversion efficiencies of 1-D nanostructures by introducing 0-D NPs. Nevertheless, the current reports mainly focused on vertically aligned ZnO nanorod and nanowire arrays, and less attention was paid to other 1-D nanostructures. On the other hand, the synthesis of the composite layer generally involves a multistep process, i.e., first formation of ZnO seeds layer on the substrate, followed by the growth of ZnO NWs array on ZnO seeding/substrates through hydrothermal methods, and eventually followed by introducing ZnO NPs between these NWs which, however, not only increases the instruments cost but also restricts the possibility of delicately tuning the concrete state of NPs and/or 1-D stuctures. For these reasons, simpler and controllable synthesis methods allowing direct fabrication of ZnO composite nanostructures are highly desirable for DSCs applications.

Herein, we report a simple one-step solvothermal method for the preparation of innovative ZnO hybrid architecture composed of 1-D ZnO NRs and 0-D ZnO NPs, in which ZnO NPs could offer large surface area for dye absorption while 1-D ZnO NRs could provide direct electron transport highway and act as light-scattering centers. A key feature of the intentionally designed synthesis route is to tune synchronously the connectivity and dispersion of ZnO NPs and NRs by controlling the reaction temperature. The obtained ZnO materials were then used to construct photoanodes of DSCs, and the photovoltaic tests revealed that the efficiencies were strongly dependent on the reaction temperature and film thickness. Under an optimized condition, a maximum efficiency of 4.30% was achieved with the photoanode assembled with 14.7 µm-thick ZnO composite synthesized at 160 °C. Various measurements, including ultaviolet–visible (UV−vis) absorption and electrochemical impedance spectroscopy (EIS), ascribed the optimum performance to the excellent fast electron transport combined with large surface area and strong light scattering.

## 2. Experimental Section

### 2.1. Materials

Anhydrous lithium iodide (LiI, 99.5%), tert-butylpyridine (t-BPy, 96%), iodide (I_2_, 99%), 3-methoxypropionitrile (99%), acetonitrile (99.9%), 2,3-dimethyl-1-propyl imidazolium iodide (DMPII, 99%), and chloroplatinic acid (H_2_PtCl_6_, 99.9%) were all received from Sigma. Diethylene glycol ((HOCH_2_CH_2_)_2_O), zinc acetate dehydrate ((CH_3_COO)_2_Zn, 99.0%), and anhydrous ethanol were all used as purchased from commercial sources without further treatment. Ru-based N719 dye [cis-bis(isothiocyanato)bis-(2,2-bipyridyl-4,4-dicarboxylato) ruthenium (II) bis(tetrabutyl-ammonium)] was provided by dyesol (New South Wales, Australia).

### 2.2. Preparation of ZnO Hybrid Nanostructures

Various ZnO architectures were synthesized via a simple polyol-mediated solvothermal approach, similar to the method reported by Cao et al. [30]. For example, the synthetic procedure of ZnO at 160 °C was performed by adding 1.416 g zinc acetate dihydrate to a 100 mL Teflon liner containing 60 mL diethylene glycol, followed by transfering the mixture into a stainless steel autoclave. After heating for 8 h at 160 °C in an oven without stirring, the as-obtained colloidal suspension was centrifuged at 8000 rpm for 15 min to deposit ZnO from the above solvent, followed by repeating centrifugation-dispersion with ethanol several times. The collected precipitate was finally dried at 60 °C for 8 h at ambient condition and subsequently grinded for further use. In parallel, other three ZnO samples prepared at the temperatures of 120 °C, 140 °C, and 180 °C were obtained following the same precedure as mentioned above. For simplicity, all four samples are referred to as Z1, Z2, Z3, and Z4 successively with the increasing order of temperature used for synthesis.

### 2.3. Preparation of ZnO Electrodes and Dye-Sensitized Solar Cells (DSCs)

A viscous ZnO paste was synthesized by mixing the as-prepared ZnO with ethyl cellulose and terpineol at weight ratio of 1:0.5:3, followed by continuous grinding for approximately 1 h [9]. Prior to fabrication of ZnO photoanode, the substrate, namely FTO conducting glass (Nippon Sheet Glass (NSG), 7 Ω/sq) was ultrasonically cleaned sequentially in HCl, acetone, and ethanol for 15 min each. Afterwards, the obtained ZnO colloid was spread on FTO substrate using a typical doctor-blading method to form a photoanode film with an active area of 0.16 cm^2^. After being allowed to dry, the ZnO films were treated in programmed heating of 325 °C for 5 min, then 375 °C for 5 min, stepwise to 450 °C for 15 min, and finally 500 °C for 15 min for the sake of removing the residual components and also strengthening the contact between FTO substrate and film, as well as that between ZnO products. The control over the ZnO film thickness was achieved by adjusting the layer of the adhesive tape, yielding ZnO photoanode films with thicknesses of 9.8, 14.7 and 20 μm.

The working electrodes were prepared by soaking the ZnO films in 0.5 mM ethanolic solution of N719 dye for 60 min at room temperature followed by careful rinsing to remove unabsorbed dye from the surface. The corresponding counter electrodes were prepared by drop-casting of H_2_PtCl_6_ solution (0.35 mM) on FTO substrate followed by annealing at 400 °C for 30 min. The sensitized ZnO photoanode and Pt-coated photocathode were then assembled to fabricate an open sandwich cell with 60 µm-thick surlyn sheets as separating layer. A drop of I^−^/I_3_^−^ -based electrolyte (0.12 M I_2_, 1.0 M DMPII, 0.5 M t-BPy and 0.1 M LiI in 3-methoxypropionitrile) was added from one side to fill the whole internal space between the two electrodes by vacuum backfilling.

### 2.4. Material Characterization and Photoelectrochemical Measurements

The crystal structures of the ZnO powders were identified by X-ray diffractometer (XRD, X’Pert PRO MPD, Panalytical, Almelo, Netherlands). The morphologies were examined by field emission scanning electron microscope (FE-SEM, SU8000, Hitachi, Tokyo, Japan) and transmission electron microscopy (TEM, JEOL-2010, Hitachi, Tokyo, Japan and Talos F200 X, Thermo Fisher, Massachusetts, USA). The specific surface areas of the materials were acquired from Brunauer–Emmett–Teller (BET) N_2_ adsorption–desorption (Autosorb iQ-XR Analyzer, Quantachrome Instruments, Boynton Beach, FL, USA). The measurement of the dye loading amount on photoanode surfaces was performed by fully desorbing the anchored dye from sensitized ZnO films into mixed solution of water and ethanol (50:50, *v*/*v*) containing 1.0 M NaOH, following the quantitative analysis with UV–vis spectrophotometry (Cary 500, Varian, Palo Alto, CA, USA). The incident photon-to-current conversion efficiency (IPCE) values and reflectance properties of the samples were investigated by a PV measurements QEX10 instrument with the wavelength range of 200–800 nm. The performances of assembled solar cells were evaluated by a photocurrent-voltage (J–V) analysis instrument (PV Measurements, IV5, Newport Corporation, Los Angeles, CA, USA) coupled with a class solar simulator. Prior to specimen measurement, calibration of the solar-simulated light intensity was conducted by means of NREL calibrated Si solar cell (PV Measurements, Inc., Boulder, CO, USA). The electrochemical impedance spectroscopy (EIS) analyses of DSCs were obtained on an electrochemical workstation (CHI760, CH Instruments, Shanghai, China). An open-circuit voltage and AC amplitude of 10 mV were applied during EIS measurements and frequencies ranging from 0.1 to 10^5^ Hz.

## 3. Results and Discussion

### 3.1. Morphological Characterization

**Figure 1:**
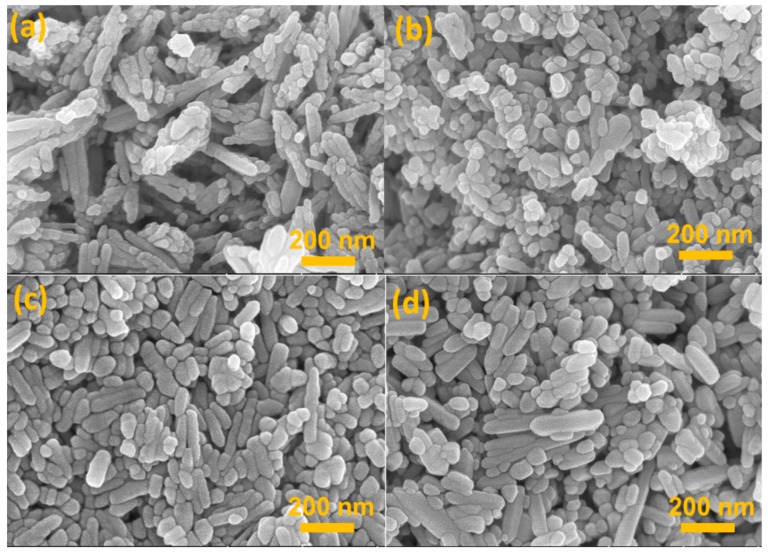
Scanning electron microscope (SEM) images of (**a**) Z1, (**b**) Z2, (**c**) Z3, and (**d**) Z4, respectively.

**Figure 2:**
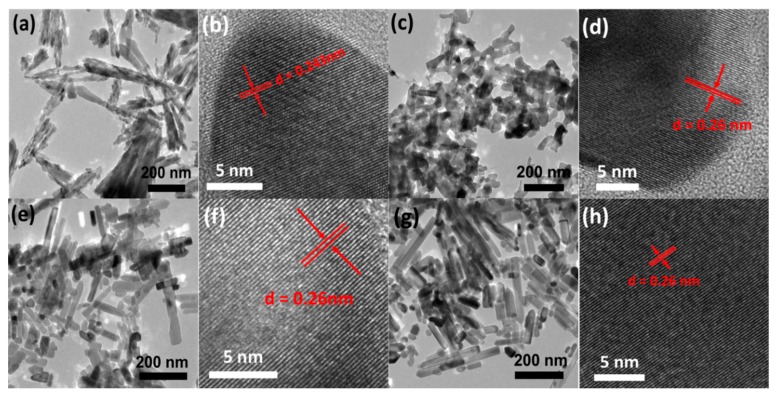
Transmission electron microscope (TEM) image and high-resolution (HRTEM) image of as-prepared ZnO samples: (**a**,**b**) Z1, (**c**,**d**) Z2, (**e**,**f**) Z3, and (**g**,**h**) Z4, respectively.

Figure 1 clearly show the typical SEM images of all these ZnO samples, where Figure 1a–d are corresponding to the products that belong to Z1 through Z4, respectively. As shown in Figure 1a and Figure 2a, the ZnO sample prepared at 120 °C (i.e., Z1) is mainly composed of a fan-shaped bundle assembled by orderly aligned NRs with lengths less than 300 mm. It is worth noting that the morphology of the constitutional unit looks quite different, with one side of the NR displaying flat and smooth surface with a typical hexagonal edge while the other end assembled by connected NPs showing a rough surface (Figure S1a). The magnified TEM image in Figure S1b revealed distinct diameter difference in single NR, changing from 10 nm at the bottom to about 30 nm at the tip. Apart from the part involved in the fan-shaped bundle, other NPs tended to aggregate together to yield rare individual nanoparticles (Figure S1c). A further high-resolution TEM (HRTEM) image (Figure 2b) of individual NR displayed high crystallinity of the synthesized products with wurtzite hexagonal structure. Lattice fringe measurement based on the HRTEM image was also conducted, and the obtained interplanar spacing (d = 0.26 nm) was closely consistent with the adjacent d-spacing of (001) plane of ZnO [31,32]. When the reaction temperature was altered to 140 °C, the fanlike bundle vanished accompanied with the formation disordered NPs and NRs. As shown in Figure 1b, the lengths of the rods clearly reduced and morphology became more irregular when compared to that displayed in Figure 1a. More importantly, TEM image in Figure 2c clearly revealed that the newly formed ZnO were apt to adhere with each other, implying partial retention of aggregation or interconnection of the particular structure. Such a morphological feature is believed to be favorable for fast electron transport rather than dye adsorption attributed to decreased surface area (see Table 1). The single crystalline feature of Z2 was also observed in perfectly aligned lattice fringes (Figure 2d) with estimated interplane spacing around 0.26 nm, matching well with the (001) plane of wurtzite ZnO.

At elevated synthesis temperature of 160 °C, the acquired composite products presented NPs with minimum size of 15 nm and NRs with maximum length of 200 nm and uniform diameter (Figure 1c and Figure S2). Comparison with Z2 revealed a significant decrease in interconnections between NPs in Z3 sample, similar to findings of Cao et al. which noticed a gradual reduction in the degree of aggregation in primary nanocrystallites at synthesis temperatures from 160 °C to 190 °C [30]. Moreover, despite the improved dispersion of NPs and NRs in sample Z3, no obvious increase in particles size was observed. This may partially be ascribed to intrinsic nature of the employed solvent (diethylene glycol). As reported previously, the significant increase of OH^−^ in the solution will definitely result in the formation of [Zn(OH)_4_]^2−^, which is inclined to adsorb on the positively charged (0001) facet of the ZnO, thus facilitating growth along the (0001) orientation [33]. However, the nonpolar nature of diethylene glycol medium inhibited the anisotropic growth of ZnO crystals to a large extent. At synthesis temperature of 180 °C, ZnO nanostructures still comprised of NPs and NRs but most ZnO surfaces became smoother and the tips sharper when compared to the product obtained at 160 °C (Figure 1d and Figure 2g), suggesting the noticeable impact of reaction temperature on the morphologic features. Moreover, the diameters of NRs looked almost uniform along the growth direction in spite of different lengths, and seemed larger than that of Z3, which could bring about a slight sacrifice of the overall surface area of ZnO products and thus dye absorption amount, as confirmed by characteristic parameters shown in Table 1. Both HRTEM images of Z3 and Z4 (Figure 2f,h) revealed lattice spacing constant around 0.26 nm, indicating the fringes of (001) plane and suggesting preferred growth along the (0001) direction.

**Figure 3:**
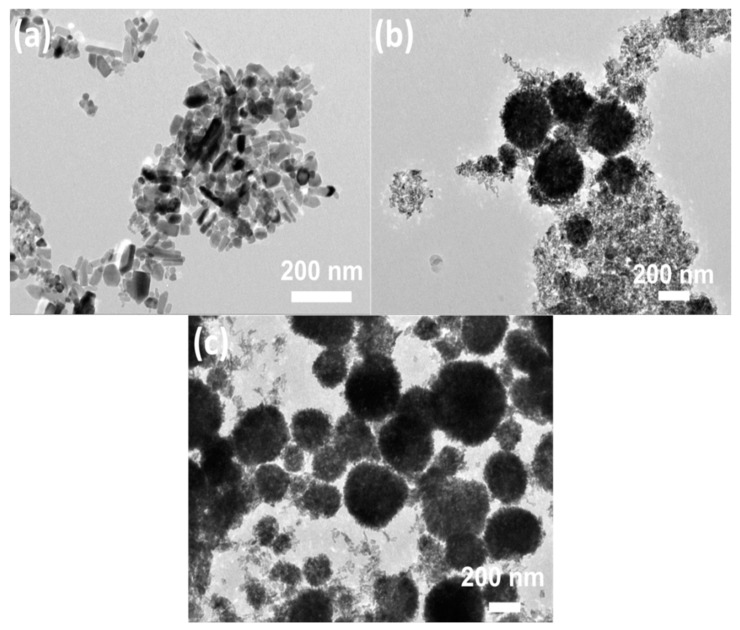
TEM images of ZnO synthesized via a thermostatic oil bath method at 160 °C under different stirring rate: (**a**) 0 r/min, (**b**) 800 r/min, and (**c**) 2000 r/min.

Compared to other reports dealing with diethylene glycol mediated synthesis route, the morphologies of our products appeared unique despite the use of same raw materials [8,28,34]. The reason for this would presumably be related to the absence of stirring during the entire synthesis process. This also induced major differences with respect to other similar studies at the exception of the reaction temperature. To confirm this assumption, ZnO specimens were also prepared by immersing the sealed stainless steel autoclave in a thermostatic oil bath at 160 °C. As can be seen in Figure 3, the amount of spherical aggregates increased gradually as stirring speed changed from low to high, confirming the pivotal role played by stirring speed on the morphology of ZnO structure. The reason for this could be attributed to the concentration gradient of Zn ions in the solvent, which would significantly be influenced by stirring. This, in turn, affected the supersaturation levels and mass transport during the reaction to some extent, leading to variation in aggregating degree and composition unit [26,35].

### 3.2. X-ray Diffraction (XRD) Analysis

**Figure 4:**
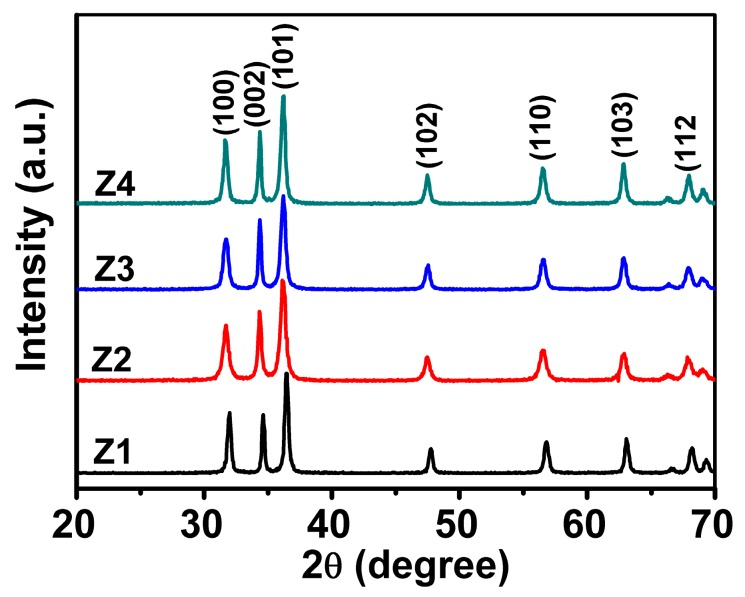
Typical X-ray diffraction (XRD) patterns of ZnO products from Z1 to Z4.

To confirm the crystal structures of the fabricated ZnO, X-ray diffraction (XRD) patterns were recorded and the results are depicted in Figure 4. The diffraction peaks of all specimens Z1–Z4 looked sharp, indicating high crystallinity. Moreover, it can be clearly observed that almost no obvious peak intensity variation is detected for all the products despite notable morphology differences, indicating the slight crystal size difference [36]. Therefore, though the reaction temperature significantly influenced the composition unit, its effect on crystallinity was almost negligible. Further observations revealed that all characteristic diffraction peaks could be well indexed to specific crystal planes of wurtzite hexagonal structured ZnO (JCPDS No. 36-1451). On the other hand, no diffraction peaks belonging to the secondary phase or impurities were detected, implying the high purity of the fabricated ZnO nanostructures [37]. By comparing with other peaks, it can be easily visualized that the full width of the (002) peak is relatively narrower, probably indicating that the growth along the (0001) direction is preferred for the as-formed ZnO crystal, [38] which is consistent with TEM image shown in Figure 2.

### 3.3. Photovoltaic Performance Test

**Figure 5:**
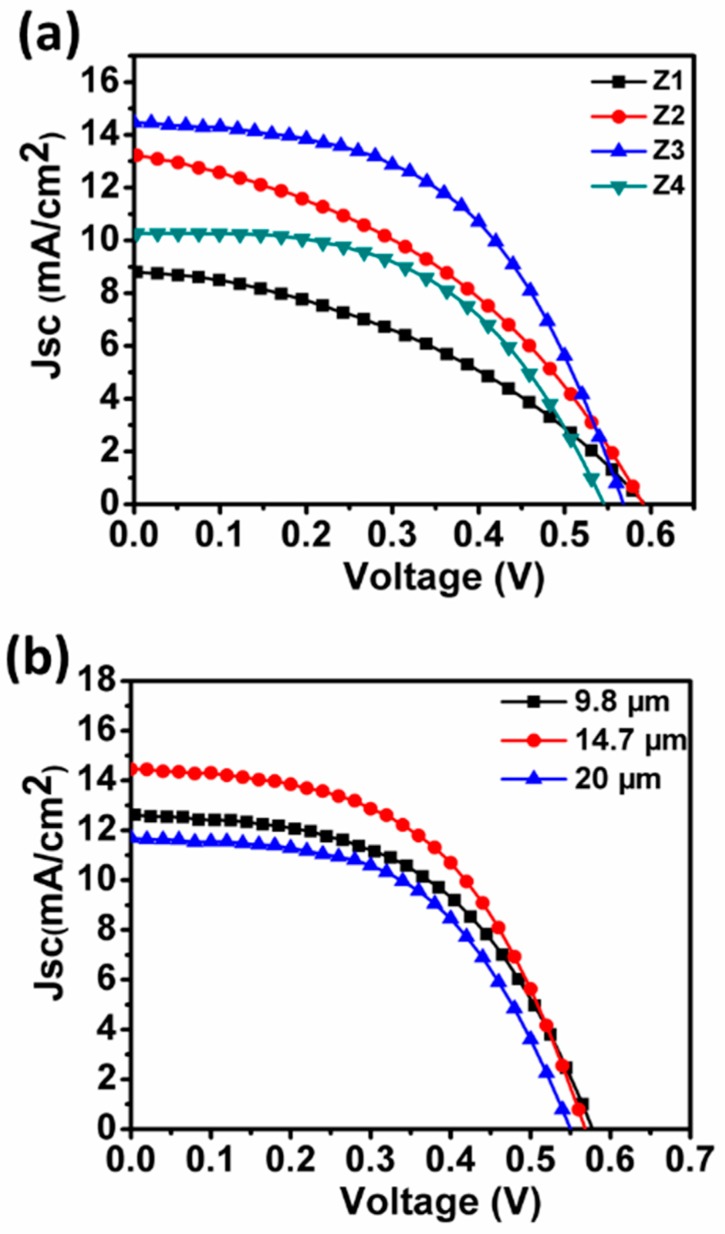
(**a**) J−V characteristic curves of various dye-sensitized solar cell (DSC) devices and (**b**) J−V characteristic curves of Z3-based solar cells as a function of film thickness.

To evaluate the photovoltaic properties of the as-prepared ZnO, four types of DSCs based on samples Z1–Z4 were constructed, and current versus voltage (J–V) behaviors were tested under illumination of AM 1.5 simulated sunlight (100 mW·cm^−2^). Typical J–V curves and calculated functional parameters, including open-circuit voltage (Voc), short-circuit current density (Jsc), fill factor (FF), and PCE are gathered in Figure 5a and Table 1. As shown in Figure 5a, the obtained Jsc, Voc, and PCE varied among Z1 to Z4. Typically, Z1 with fan-shaped nanostructure achieved Jsc of 8.72 mA·cm^−2^, Voc of 0.593 V, and PCE of 2.07%. Note that conversion efficiencies ranging from 1.5% to 2.7% were generally achieved by DSCs based on NRs and NWs, indicating the inherent limitation of formed architecture [39,40,41]. The increase in temperature to 140 °C and 160 °C remarkably raised Jsc of Z2- and Z3-based DSCs to yield values reaching 13.30 mA·cm^−2^ and 14.50 mA·cm^−2^, respectively. By contrast, Voc of Z3 and Z4 showed reverse trends, with a decline in values to 0.591 V and 0.567 V, respectively. Afterward, the current density showed an abrupt decrease to 10.28 mA·cm^−2^ as reaction temperature increased to 180 °C. Therefore, Z3 with maximum Jsc had the highest conversion efficiency (4.30%) among all products under identical conditions. Such a relationship between the photovoltaic performance and reaction temperature further confirmed the difference in morphology and structure among Z1–Z4. For a given DSC system, the current density would most definitely be determined by the synergy of light harvesting and electron transport properties of the resultant film. Hence, the dye loading amount, optical properties and electron transport capabilities were systemically studied to clarify probable mechanisms underlying discrepancies in photovoltaic performances.

The dependence of Jsc and PCE on the thickness of Z3 was also systematically investigated to effectively combine the energy harvesting with electron transport of the photoanode configuration. As shown in Figure 5b, the current density first increased from 12.7 to 14.50 mA·cm^−2^ with film thickness from 9.8 to 14.7 µm and then drastically decreased to 11.7 mA·cm^−2^ at film thickness of 20 µm. The PCE of corresponding specimens with different thicknesses were recorded as 3.74%, 4.30% and 3.44%, respectively. No linear relationship between Jsc or PCE and thickness of ZnO photoanode was noticed, which might be ascribed to competing effects between the enhanced light harvesting arising from the increased dye adsorption and charge recombination within the resultant film, both believed to be closely related to photoanode thickness [9,42].

### 3.4. Dye Absorption and Diffuse Reflectivity

To explore the origin of the obvious difference in photovoltaic performance between samples, the dye-loading capabilities of ZnO films with various structures were studied and the corresponding results are listed in Table 1. The attached dye amounts of Z1, Z2 and Z3 were estimated to be 0.87 × 10^−7^, 1.35 × 10^−7^ and 2.15 × 10^−7^ mol·cm^−2^, respectively. This confirmed the gradual increasing trend with temperature from 120 to 160 °C. However, Z4 depicted an ultimate decrease in dye loading amount (1.41 × 10^−7^ mol·cm^−2^). Since all photoanode films had the same thickness, the significant discrepancy in dye absorption must be due to the difference in overall surface area between samples. The latter was confirmed by BET specific surface area (S_BET_) measurements provided in Table 1. Z3 presented the highest surface area of 24.4 m^2^·g^−1^, whereas Z1 revealed the lowest value of 9.8 m^2^·g^−1^ followed by Z2 and Z4, displaying the same variation trend as the amount of absorbed dye. Higher dye loading amount makes it possible for sufficient absorption of incident photons, thus providing the possibility of achieving larger Jsc and PCE.

**Figure 6:**
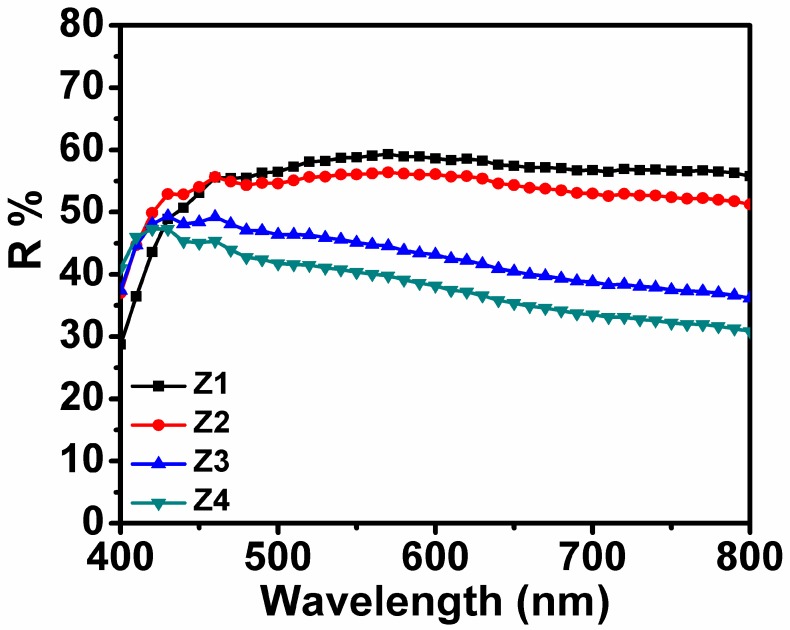
Diffuse reflectance spectra of all four photoanode films from Z1 to Z4.

To estimate the light-scattering properties of resultant ZnO photoanode films, the diffuse reflectance spectra were further investigated and the data are provided in Figure 6. The reflectance values of Z1 and Z2 films were apparently higher than those of Z3 and Z4 over the wavelength range of 450–800 nm. Hence, films constructed with fan-shaped NRs and partially aggregated NRs and NPs had higher reflecting abilities than the other two films assembled with relatively dispersed NRs and NPs. This was understandable since aggregation or assembling of NRs and/or NPs did not only favor the formation of large particles with size comparable to the wavelength of incident light but also benefited formation of voids, resulting in enhanced light scattering and decreased light transmittance of the incident light. In addition, the reflectivity difference between Z1 and Z2 might also be associated with the degree of aggregation or arranging pattern of the fundamental composition units (NRs and NPs). By comparison, the distinction between Z3 and Z4 could mainly be attributed to the partial retention of tight connection between NRs and NPs in Z3 (Figure 2). Higher light-scattering capacities would mean more trapped and utilized light, resulting in improved electron generation within the photoanode film and enhanced Jsc. However, since Z3 presented considerably lower light scattering ability, its highest Jsc value for Z3 is most likely attributed to the increased light harvesting arising from the larger surface area-induced efficient dye absorption rather than light reflecting and scattering within the film.

### 3.5. Electrochemical Impedance Spectroscopy (EIS) Analysis

**Figure 7:**
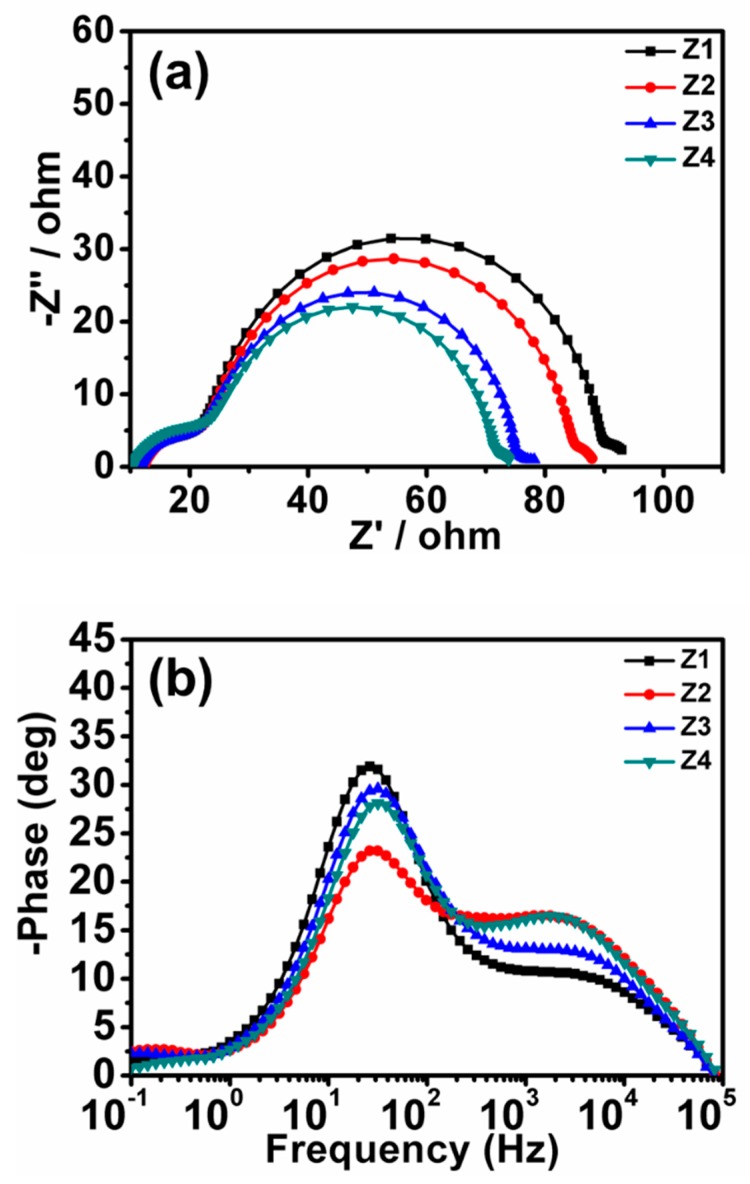
(**a**) Nyquist plots and (**b**) Bode phase plots of the ZnO cells.

To gain a deeper understanding of charge transfer behaviors of ZnO DSCs, electrochemical impedance spectroscopy (EIS) was performed at applied bias of Voc in the dark. In Figure 7a, two distinguishable semicircles were clearly observed in the EIS Nyquist plot. The first was related to redox reactions at the Pt/electrolyte interface while the second was associated with charge transfer processes occurred at oxide semiconductor film/dye/electrolyte interface. In general, information related to the electron transport properties of semiconductor films can be obtained directly from the Nyquist plot [13,43,44]. In particular, the extent of charge recombination could visually be judged from the diameter of the second central arc at medium frequencies. The central arc diameter in Nyquist plots decreased in the following sequence: Z1 > Z2 > Z3 > Z4, implying the gradually decreased recombination resistance from Z1 to Z4. Compared to Z3 and Z4, the special 1-D structure in Z1 and effective connectivity between NPs and NRs in Z2 could effectively reduce the electron trapping sites (defects, grain boundaries, and surface states) in the photoanode films to facilitate the electron transport. The elevated resistance of Z3 could be explained by morphology. Even though Z3 presented smaller sized NPs and shorter length NRs when compared to Z4, the existence of partially interconnected NPs and NRs may play a key role in decreasing possible charge recombination. The electrons’ lifetime (*τ_e_*) values in various ZnO films were estimated according to the formula: *τ_e_* = 1/ω*_max_ =* 1/2π*f_max_*, where *f_max_* is the maximum frequency of mid-frequency peaks in Bode phase plots (Figure 7b). The τ_e_ values were recorded as 6.2 ms for Z1, 5.3 ms for Z2, 4.7 ms for Z3, and 4.1 ms for Z4. The latter further demonstrated the dependence of electron transport on morphology characteristics. The much longer electron lifetimes signifies a faster diffusion rate of electrons in the films, matching the improved overall efficiencies.

### 3.6. Incident Photon-to-Current Conversion Efficiency (IPCE) Measurements

**Figure 8:**
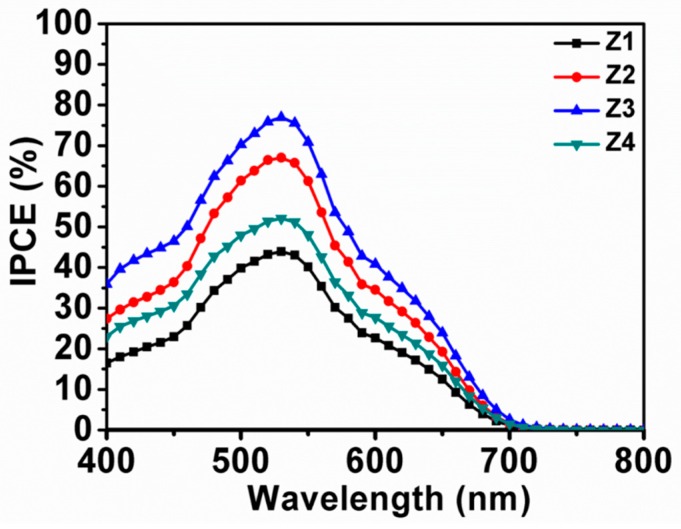
Incident photon-to-current conversion efficiency (IPCE) spectra of devices with the photoanode films constructed with Z1 to Z4.

Figure 8 compares the IPCE values of cells constructed with various samples. Clearly, Z3 based cell provided the highest IPCE value among all devices, followed by Z2, Z4 and Z1, consistent with the trend of Jsc (Figure 5). IPCE would be related predominantly to the efficiencies of sunlight harvesting, as well as electron injection and collection. Since the same sensitizer (N719) and iodine-based electrolyte were applied to all ZnO based cells, the change in IPCE can be attributed mainly to variation in light harvesting efficiency and/or charge collection efficiency [8,9,13]. These features would originate from dye absorption and light scattering capabilities, as well as electron transport properties. As revealed by the UV-vis absorption and EIS spectra, Z3 showed higher dye attachment, enhanced light harvesting, and improved electron transport properties when compared to Z4. Therefore, much higher IPCE of Z3 with respect to Z4 could be ascribed to the synergic effects of the aforementioned favoring factors. For Z1 and Z2, the decreased IPCE values could be ascribed to the reduced dye absorption abilities of the resultant photoanode films despite their higher light-scattering capabilities and larger recombination resistances.

## 4. Conclusions

In summary, a facile one-pot solvothermal method was successfully developed for the synthesis of a class of novel ZnO architectures composed of 1-D NRs and 0-D NPs. Fine tuning morphology and structure of ZnO were realized by controlling the experimental parameters, such as reaction temperature and stirring rate. This made it feasible to optimize the photovoltaic performances by maximizing the required characteristics (large dye-loading amount, excellent light scattering, and fast electron transport) of the photoelectrode. As a consequence, a significantly improved PCE of 4.30% was achieved for the sample synthesized at 160 °C (i.e., Z3) under an optimized film thickness of 14.7 μm. The material and device characterizations revealed that 0-D ZnO NPs played a pivotal role in improving the specific surface area for abundant dye absorption, while locally tight interconnection between 1-D NRs and 0-D NPs were favorable for fast electron transport and excellent light scattering. Overall, the proposed approach offered a feasible way for outstanding cell efficiencies by directional control of ZnO architecture, which should be applicable for the design of other photoanode materials of DSCs.

## Figures and Tables

**Table 1:** Characteristics of various ZnO photoanodes together with the corresponding photovoltaic parameters.

Sample	Jsc (mA/cm²)	Voc (V)	FF	PCE	Adsorbed Dye (×10^−7^ mol·cm^−2^)	S_BET_ (m^2^/g)	Crystal Size (nm)
Z1	8.72	0.593	0.400	2.07	0.87	9.8	21.5
Z2	13.30	0.591	0.405	3.18	1.35	15.4	19.8
Z3	14.50	0.567	0.523	4.30	2.15	24.4	20.6
Z4	10.28	0.545	0.556	3.12	1.41	16.6	22.8.

## Supplementary Materials

The following are available online at https://www.mdpi.com/2079-4991/9/7/931/s1, Figure S1: The TEM image of (a) magnified fan-shaped bundles, (b) aggregated NRs, and (b) aggregated NPs in Z1 recorded from Talos F200 X. Figure S2: Magnified TEM image of Z3, Figure S3: The error bars of the key photovoltaic parameters.
