# Supplementary material for: Design of Morphology-Controllable ZnO Nanorods/Nanopariticles Composite for Enhanced Performance of Dye-Sensitized Solar Cells
Source: Nanomaterials (Basel). 2019 Jun 28;9(7):931. doi: 10.3390/nano9070931 (PMC6669986; doi:10.3390/nano9070931)
Supplement: Supplementary file 1 [file nanomaterials-09-00931-s001.pdf]

Design of Morphology-Controllable ZnO Nanorods/Nanopariticles Composite for High Efficiency Dye-Sensitized Solar Cells

Dongting Wang, [a*] **Yuting Zhang,** [a] **Meng Su,** [a] **Ting Xu,** [a] **Haizhou Yang,** [a] **Shiqing Bi,** [b] **Xianxi Zhang,** [a] **Yuzhen Fang,** [a] **Jinsheng Zhao** [a]

a School of Chemistry and Chemical Engineering, Liaocheng University, Shandong Province, Liaocheng, 252059, PR China

b School of Chemistry and Chemical Engineering, Yulin University, Shaanxi Province, Yulin 719000, P. R. China

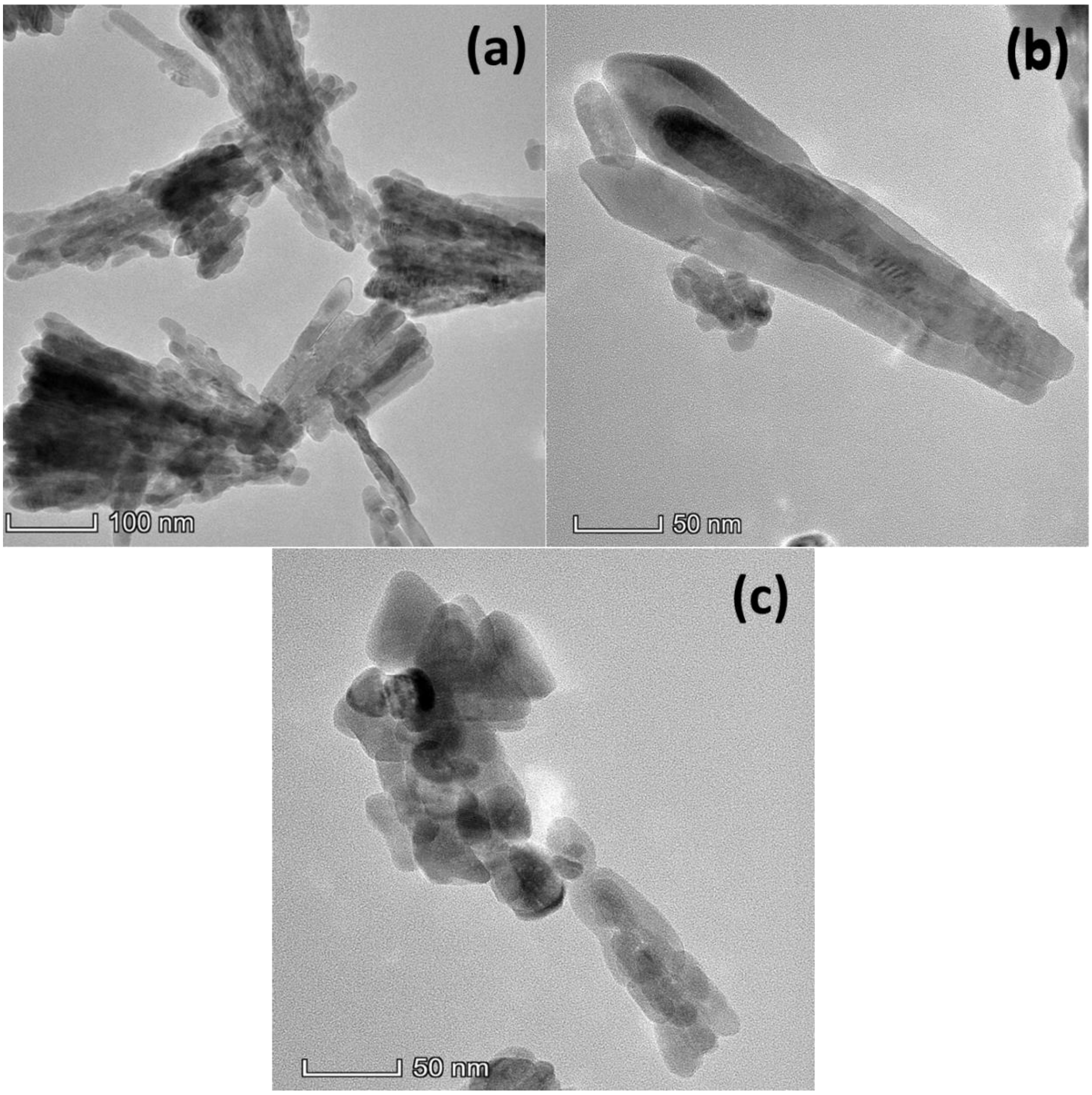

Figure S1. The TEM image of (a) magnified fan-shaped bundles, (b) aggregated NRs, and (c) aggregated NPs in Z1 recorded from Talos F200 X.

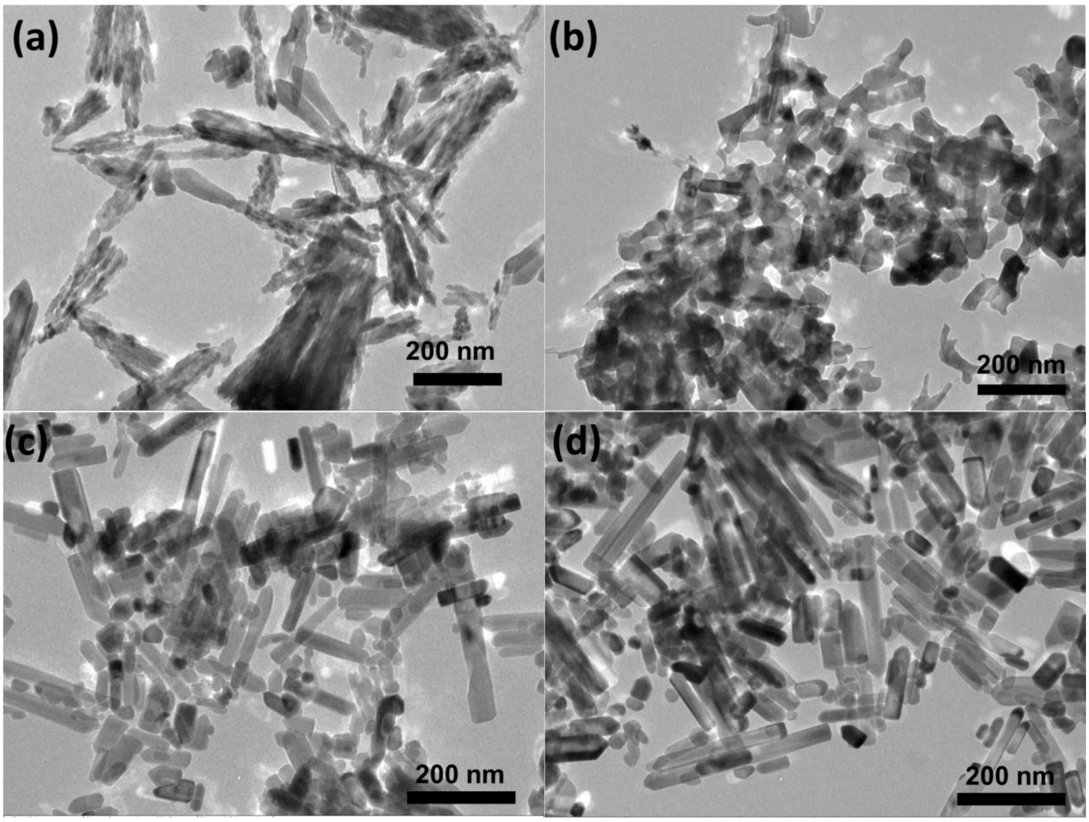

Figure S2. The TEM images of (a) Z1, (b) Z2, (c) Z3 and (d) Z4, respectively, recorded from JEOL-2010.

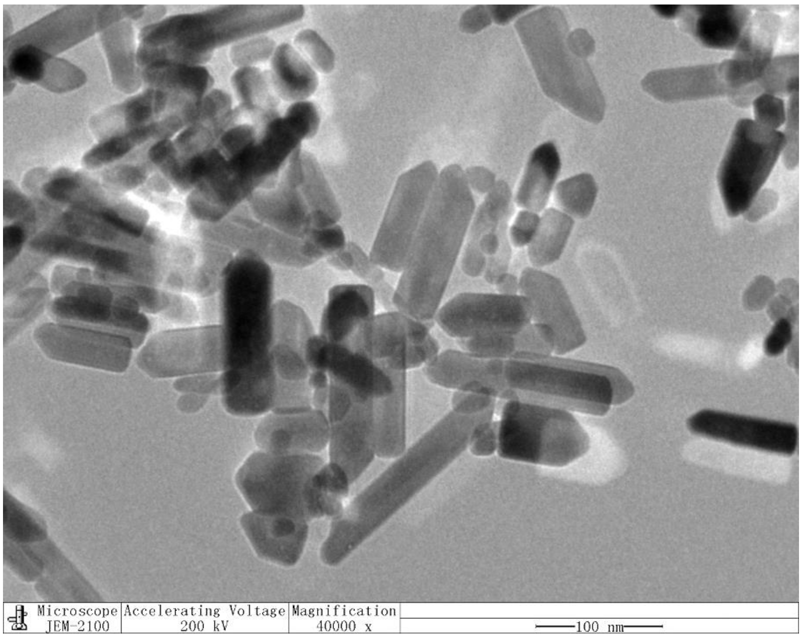

Figure S3. Magnified TEM image of Z3.
